# Reduced serum levels of ACTBL2, CADH7, and PKMYT1 in Bipolar Disorder

**DOI:** 10.1192/j.eurpsy.2026.11214

**Published:** 2026-07-31

**Authors:** T. Amstislavskaya, D. Kazantseva, A. Seregin, L. Smirnova, S. Ivanova

**Affiliations:** 1 Scientific Research Institute of Neurosciences and Medicine, Novosibirsk; 2 Laboratory of Molecular Genetics and Biochemistry, Mental Health Research Institute Tomsk National Research Medical Center Russian Academy of Sciences, Tomsk, Russian Federation

## Abstract

**Introduction:**

Bipolar disorder (BD) is a chronic psychiatric condition characterized by alternating episodes of hypomania and depression, leading to significant social consequences. Differential diagnosis remains challenging due to clinical overlap with other psychiatric disorders and the absence of paraclinical diagnostic biomarkers, reflecting insufficient understanding of its pathophysiological mechanisms. We investigated ACTBL2, CADH7, and PKMYT1 as biologically relevant candidate proteins given their roles in synaptic structure, neuronal adhesion, and genomic integrity—processes that are increasingly implicated in the development of BD.

**Objectives:**

To investigate serum levels of three proteins—ACTBL2 (actin-like beta-2), CADH7 (cadherin-7), and PKMYT1 (protein kinase, membrane associated tyrosine/threonine 1)—in patients with BD compared to healthy controls.

**Methods:**

Patients with BD in a mixed episode (median age 37 years, IQR [25;41]; illness duration 7 years [4;11]), assessed using the SIGH-SAD and CGI scales, were compared with mentally and physically healthy volunteers (median age 30 years [25;39]). Serum protein levels were quantified by immunoblotting: proteins were transferred to PVDF membranes and probed with primary antibodies against ACTBL2, CADH7, and PKMYT1 (Affinity Biosciences, China). Detection and densitometric analysis were performed using the iBright FL1500 Imaging System (Thermo Scientific, USA) at the Shared Research Center “Medical Genomics,” Tomsk National Research Medical Center. Group differences were evaluated using the Mann–Whitney U test (p < 0.05) in Statistica 10.0.

**Results:**

Significantly lower serum levels of all three proteins were observed in BAD patients versus controls: ACTBL2 (p = 0.032), CADH7 (p = 0.043), and PKMYT1 (p = 0.047).

**Conclusions:**

The reduced levels of ACTBL2, CADH7, and PKMYT1 may reflect core neurobiological disturbances in BAD—namely, impaired synaptic and cytoskeletal dynamics, weakened interneuronal adhesion leading to disrupted network coherence, and compromised maintenance of genomic and cellular homeostasis. While preliminary, these findings suggest that the three proteins warrant further investigation as potential paraclinical biomarkers for BAD.

Support by the Russian Science Foundation grant No. 23-75-00023.

**Disclosure of Interest:**

None Declared

